# Rethinking Autism as an Affective-Empathy Disorder

**DOI:** 10.31083/AP45867

**Published:** 2026-02-03

**Authors:** Sergio Machado, Flávia Paes, João Lucas Lima

**Affiliations:** ^1^Center for Neuroscience, Neurodiversity Institute, 26210-070 Nova Iguaçu-RJ, Brazil; ^2^Panic and Respiration Laboratory (LABPR), Institute of Psychiatry (IPUB), Federal University of Rio de Janeiro (UFRJ), 22290-140 Rio de Janeiro, Brazil; ^3^Virtual Reality in Mental Health Laboratory (RVSM), Institute of Psychiatry (IPUB), Federal University of Rio de Janeiro (UFRJ), 22290-140 Rio de Janeiro, Brazil

Autism spectrum disorder (ASD) has traditionally been understood through the lens of deficits in Theory of Mind (ToM), which emphasizes challenges in inferring the mental states of others [1]. However, emerging research has suggested a paradigm shift: the core of autism may lie in affective empathy, specifically in a disruption of intercorporeality, which is the pre-reflective, bodily connection that underpins our ability to attune to others emotionally [2, 3].

Although ToM is useful for explaining certain cognitive limitations in ASD, it fails to address the disorder’s early signs. As Schnitzler and Fuchs [2] pointed out , autistic children often exhibit reduced social smiling, spontaneous imitation, and eye contact before the age of two. These are markers that precede the development of ToM, which typically emerges around four years of age [4, 5]. Furthermore, ToM is rooted in a Cartesian view that separates mind and body, treating emotions as inaccessible internal entities to be deciphered through external cues. This perspective overlooks the embodied nature of empathy, which arises from direct bodily resonance rather than inference [6, 7].

Schnitzler and Fuchs [2] argued that ASD is, above all, a disorder of intercorporeality. Studies involving infants later diagnosed with autism revealed atypical physiological responses to the distress of others, such as reduced heart-rate variability [8] and decreased zygomaticus major activity (associated with smiling) when observing emotional facial expressions [9]. These anomalies, emerging prior to the capacity for mentalization, suggest a primary impairment in affective empathy, that is, the ability to feel with another, not just understand them. Phenomenologically, autistic individuals often describe a sense of bodily disconnection during social interactions [2].

This perspective carries significant implications. First, it challenges interventions that prioritize cognitive training of social skills, such as recognizing facial expressions or adhering to social rules. Instead, therapies promoting bodily synchronization, such as Greenspan’s DIR/Floortime model [10], which emphasizes emotional engagement through play, may prove more effective in rebuilding the foundations of affective empathy. Second, this perspective redefines the notion of “social deficit” in autism, which is not merely a failure to understand others, but a lack of resonance with them. This aligns with the “double empathy problem” [11], which posits that connection difficulties are bidirectional, stemming from divergent communication styles between neurodivergent and neurotypical individuals. Finally, this approach challenges stigmas. If affective empathy in autism is atypical but not absent, as a study shows intense emotional responses to specific stimuli have suggested [12], the narrative of “emotional coldness” collapses. Rather than pathologizing, we must create spaces that value alternative forms of connection, whether through art, movement, or assistive technologies that translate emotions into accessible sensory languages.

In summary, autism should be reconceptualized not as a “deficit” in mind-reading, but as a difference in the embodied experience of the social world. This perspective necessitates that psychiatry embrace more holistic models in which body, emotion, and cognition are inseparable. As Merleau-Ponty described [6], the body is our general means of having a world; for autistic individuals, this world may resonate differently, yet it remains profoundly human.
